# Hesperidin, a citrus bioflavonoid, decreases the oxidative stress produced by carbon tetrachloride in rat liver and kidney

**DOI:** 10.1186/1471-2210-5-2

**Published:** 2005-01-31

**Authors:** Naveen Tirkey, Sangeeta Pilkhwal, Anurag Kuhad, Kanwaljit Chopra

**Affiliations:** 1 Pharmacology division, University Institute of Pharmaceutical Sciences, Panjab University, Chandigarh-160014, India

## Abstract

**Background:**

CCl_4 _is a well-established hepatotoxin inducing liver injury by producing free radicals. Exposure to CCl_4 _also induces acute and chronic renal injuries. The present study was designed to establish the protective effect of hesperidin (HDN), a citrus bioflavonoid, on CCl_4_-induced oxidative stress and resultant dysfunction of rat liver and kidney.

**Methods:**

Animals were pretreated with HDN (100 and 200 mg/kg orally) for one week and then challenged with CCl_4 _(2 ml/kg/s.c.) in olive oil. Rats were sacrificed by carotid bleeding under ether anesthesia. Liver enzymes, urea and creatinine were estimated in serum. Oxidative stress in liver and kidney tissue was estimated using Thiobarbituric acid reactive substances (TBARS), glutathione (GSH) content, superoxide dismutase(SOD), and Catalase (CAT)

**Results:**

CCl_4 _caused a marked rise in serum levels of ALT and AST (P < 0.05). TBARS levels were significantly increased whereas GSH, SOD and CAT levels decreased in the liver and kidney homogenates of CCl_4 _treated rats. HDN (200 mg/kg) successfully attenuated these effects of CCl_4_

**Conclusion:**

In conclusion, our study demonstrated a protective effect of HDN in CCl_4 _induced oxidative stress in rat liver and kidney. This protective effect of HDN can be correlated to its direct antioxidant effect.

## Background

Drug exposure, ionizing radiations and environmental pro-oxidant pollutants induce free radical formation. Lipid peroxidation initiated by free radicals is considered to be deleterious for cell membranes and has been implicated in a number of pathological situations. Carbontetrachloride (CCl_4_), an industrial solvent, is a well-established hepatotoxin [1-3]. Various Studies demonstrated that liver is not the only target organ of CCl_4 _and it causes free radical generation in other tissues also such as kidneys, heart, lung, testis, brain and blood [4-6]. It has also been reported that exposure to CCl_4 _induces acute and chronic renal injuries [7,8]. Case control studies and various documented case reports increasingly establish that hydrocarbon solvents produce renal diseases in humans [9].

Extensive evidence demonstrates that as a result of the metabolic activation of CCl_4_, ^•^CCl_4 _and ^•^Cl, are formed which initiate lipid peroxidation process. Vitamin E protected CCl_4_-induced liver injury indicating the role of oxidative stress in this model [10]. Studies also show that certain natural extracts containing antioxidants protect against the CCl_4_-induced increased lipid peroxide levels and impairment in hepatic GSH status [11].

Hesperidin is a flavanone glycoside abundantly found in sweet orange and lemon and is an inexpensive by-product of citrus cultivation [12]. Hesperidin is effectively used as a supplemental agent in the treatment protocols of complementary settings. Its deficiency has been linked to abnormal capillary leakiness as well as pain in the extremities causing aches, weakness and night leg cramps. Supplemental hesperidin also helps in reducing oedema or excess swelling in the legs due to fluid accumulation. A number of researchers have examined the antioxidant activity and radical scavenging properties of hesperidin using a variety of assay systems [13-16].

Thus the present study was designed to investigate the effect of HDN on CCl_4_-induced oxidative stress and resultant dysfunction of rat liver and kidney.

## Results

### Effect on liver enzymes

CCl_4 _caused a marked rise in serum levels of ALT (control = 45 IU/L) and AST (control = 135 IU/L) demonstrating a marked liver damage. Treatment with HDN decreases the elevated levels of ALT and AST in serum (P < 0.05)(Table 1). Both the doses of HDN also attenuated the CCl_4_-induced elevated levels of total bilirubin (control = 0.184 mg/dl).

**Table 1:** Effect of different doses of Hesperidin on CCl_4 _induced rise in AST, ALT and total bilirubin.

	**ALT (IU/L)**	**AST(IU/L)**	**Bilirubin total (mg/dl)**
**Control**	100	100	100
**CCl_4_**	255.55 ± 23.66^a^	444.44 ± 22.56^a^	205.21 ± 11.66^a^
**HDN (200)**	112.81 ± 13.66	126.66 ± 28.66	108.15 ± 9.66
**CCl_4_+HDN (100)**	185.18 ± 17.56^a,b^	333.33 ± 29.66^a,b^	176.63 ± 15.29^a,b^
**CCl_4_+HDN (200)**	144.44 ± 15.22^a,b,c^	244.44 ± 22.66^a,b,c^	149.45 ± 12.45^a,b,c^

Values are expressed as percent response compared to control rats. a = Statistical significant at P < 0.05 as compare to control, b = Statistical significant at P < 0.05 as compare to CCl_4_, c = Statistical significant at P < 0.05 as compare to CCl_4_+ HDN(100)

### Effect on hepatic and renal TBARS levels

**Figure 1:**
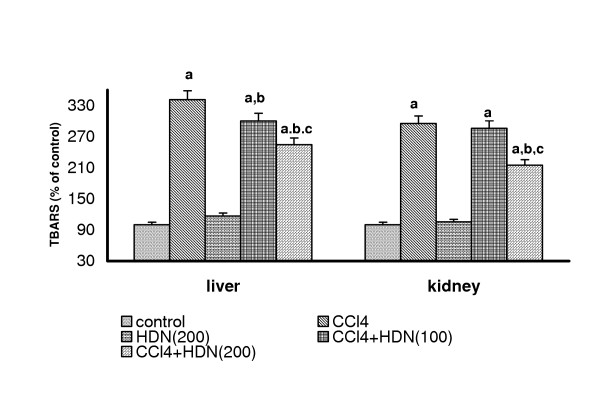
Effect of different doses of Hesperidin on CCl_4 _induced lipid peroxidation in rat liver and kidney. Values are expressed as percent response compared to control rats. a = Statistical significant at P < 0.05 as compare to control, b = Statistical significant at P < 0.05 as compare to CCl_4_, c = Statistical significant at P < 0.05 as compare to CCl_4_+ HDN(100)

CCl_4 _challenge caused a marked lipid peroxidation in both liver (control = 1.5 micromoles/mg protein) and kidney (control = 33.86 nmoles/mg protein). Both the doses of HDN decreased the level of lipid peroxidation in liver, but in the kidney, no effect on lipid peroxidation was seen with 100-mg/kg dose, and only the higher dose of HDN (200 mg/kg) could attenuate the increased level of lipid peroxidation (P < 0.05) (Fig 1). 7-day oral feeding of HDN per se (200 mg/kg) did not result in a significant alteration of either hepatic or renal TBARS levels.

### Effect on the glutathione levels in CCl_4 _treated rats

**Figure 2:**
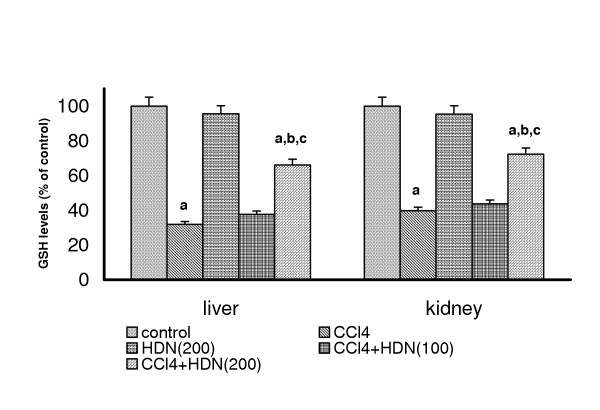
Effect of different doses of Hesperidin on CCl_4 _induced depletion in GSH levels in rat liver and kidney. Values are expressed as percent response compared to control rats. a = Statistical significant at P < 0.05 as compare to control, b = Statistical significant at P < 0.05 as compare to CCl_4_, c = Statistical significant at P < 0.05 as compare to CCl_4_+ HDN(100)

CCl_4 _administration markedly decreased the levels of reduced glutathione in both the liver (control = 35.99 micromoles/mg protein) and kidneys (control = 27.99 micromoles/mg protein) demonstrating oxidative stress. HDN (200 mg/kg) *per se *did not produce any change in the levels of reduced glutathione either in liver or kidney. HDN (100 mg/kg) showed no effect on the levels of reduced glutathione either in liver or kidney in CCl_4 _treated rats whereas HDN (200 mg/kg) significantly ameliorated CCl_4_-induced depletion of GSH in both liver and kidney (P < 0.05)(Fig-2). HDN per se (200 mg/kg) did not result in a significant alteration of either hepatic or renal GSH levels.

### Effect on the antioxidant enzymes in liver and kidneys in CCl_4 _treated Rats

**Figure 3:**
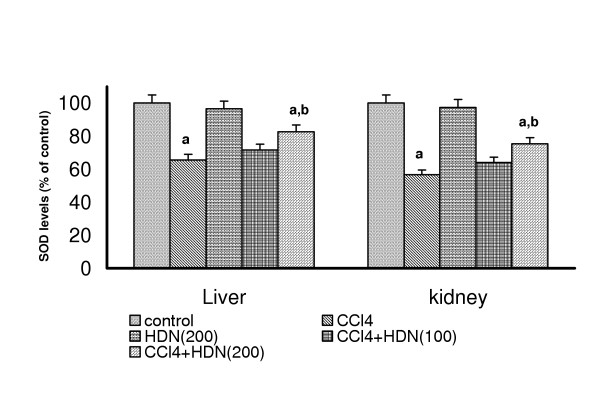
Effect of different doses of Hesperidin on CCl_4 _induced depletion in SOD levels in rat liver and kidney. Values are expressed as percent response compared to control rats. a = Statistical significant at P < 0.05 as compare to control, b = Statistical significant at P < 0.05 as compare to CCl_4_

**Figure 4:**
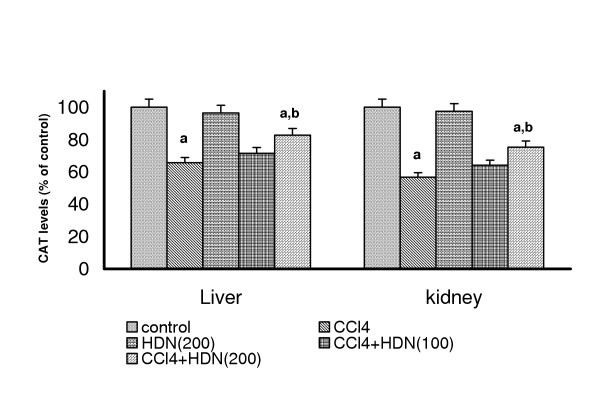
Effect of different doses of Hesperidin on CCl_4 _induced depletion in Catalase levels in rat liver and kidney. Values are expressed as percent response compared to control rats. a = Statistical significant at P < 0.05 as compare to control, b = Statistical significant at P < 0.05 as compare to CCl_4_

CCl_4 _challenge significantly decreased the levels of SOD and catalase in both liver (SOD: control = 25.66 U/mg protein; Catalase: control = 0.32 K/min) and kidneys (SOD: control = 99.22 U/mg protein; Catalase: control = 0.32 K/min). HDN per se (200 mg/kg) had no effect on these enzymes either in liver or in kidneys. HDN (100 mg/kg) failed to improve the levels of SOD or catalase either in liver or kidneys of CCl_4 _administered rats but HDN (200 mg/kg) significantly increased the levels of both enzymes in liver and kidneys of CCl_4 _treated rats (P < 0.05)(Fig-3 and 4). 7-day oral feeding of HDN per se (200 mg/kg) did not result in a significant alteration of any of these antioxidant enzymes either in liver or kidney.

## Discussion

CCl_4_-induced lipid peroxidation is highly dependent on its bioactivation to the trichoromethyl radical and trichloromethyl peroxy radical [17-19]. It is well known that CCl_4 _is activated by the cytochrome P450 system. The initial metabolite is the trichloromethyl free radical, which is believed to initate the biochemical events that ultimately culminate in liver cell necrosis[20,21]. The trichloromethyl radical can form covalent adducts with lipids and proteins, interact with O_2 _to form a tricholoromethyl peroxy radical or abstract hydrogen atoms to form chloroform [22]. Other products include conjugated dienes, lipid hydroperoxides, malonaldehyde-like substances, and other short-chain hydrocarbons [23-25]. In response to hepatocellular injury initiated by the biotransformation of CCl_4 _to reactive radicals, "activated" Kupffer cells in liver respond by releasing increased amounts of active oxygen species and other bioactive agents [26].

Protective effects of various natural products in CCl_4 _hepatotoxicity have been reported [27]. Studies done with Ginseng showed that the antioxidant property of ginsenosides contributes to protection against CCl_4 _induced hepatotoxicity in rats [28]. In the present study, CCl_4 _induced a severe hepatic damage as represented by markedly elevated levels of ALT, AST and bilirubin coupled with a marked hepatic oxidative stress. CCl_4_-induced generation of peroxy radicals and O_2_^-• ^leads to inactivation of catalase and SOD. We too observed that CCl_4 _challenge significantly decreased the levels of SOD and catalase in liver and kidney. Recently, Szymonik-Lesiuk et al [2] have shown that CCl_4 _intoxication can lead to alteration in gene expression and depletion of SOD and catalase levels in kidney and heart. Oxidative stress causes depletion of intracellular GSH, leading to serious consequences. HDN administration ameliorated the increased level of lipid peroxidation after CCl_4 _treatment. Interestingly, only the higher dose of HDN (200 mg/kg) was able to show improvement in the levels of endogenous antioxidant enzymes (SOD and catalase) and GSH in liver. Improvement of hepatic GSH levels in HDN-treated rats in comparison to CCl_4 _intoxicated rats demonstrates the antioxidant effect of HDN.

We failed to observe any effect of CCl_4 _on renal function. Neither BUN nor serum creatinine levels increased after CCl_4 _administration (data not shown). Studies by Zimmerman et al [29] also did not report any rise in BUN levels even after chronic treatment of CCl_4 _in nephrectomized rats. They found an increased frequency of glomerulosclerosis and tubulointerstitial alterations in rats with reduced renal mass on CCl_4 _administration thereby indicating nephrotoxicity on long-term CCl_4 _administration in rats. These findings raise the possibility that renal disease in man is related to hydrocarbon solvent exposure and may also be potentiated by concomitant renal disease or impaired renal function. Ogawa et al [30] also reported that chronic renal injuries and BUN elevations developed in Balb/c mice only after 12 weeks of CCl_4 _intoxication. On the contrary, we estimated the renal function just after 48 hrs of CCl_4 _challenge. Thus this brief period might not be sufficient to demonstrate any rise in serum BUN and creatinine levels. Though renal function did not alter after 48 hrs of CCl_4 _administration but even this short period of exposure led to a significant oxidative stress in kidneys. Fadhel and coworkers [31] had also reported increased levels of renal TBARS in rats after CCl_4 _exposure which could be improved by black tea extract. Similar observations were also reported with certain Indian ayurvedic Indian preparations [32].

HDN treatment has been previously demonstrated to improve GSH levels in liver and kidneys of diabetic rats and a decrease in levels of 8-hydroxydeoxyguanosine (8-OHdG), a marker of DNA fragmentation, in the urine of diabetic rats [33]. HDN in combination with Diosmin has also been shown to inhibit the reactive oxygen radicals production in Zymosan-stimulated human polymorphonuclear neutrophils [34]. Thus HDN has been shown to reduce oxidative stress in various in-vivo and in-vitro studies.

## Conclusions

In conclusion, our study demonstrated that CCl_4 _induces a marked oxidative stress in rat liver and kidney, which is amenable to attenuation by HDN. This protective effect of HDN can be correlated directly to its antioxidant property.

## Methods

### Animals

Male wistar rats (150 g–200 g), bred in the central animal house of Panjab University (Chandigarh, India) were used. The animals were housed under standard conditions of light and dark cycle with free access to food (Hindustan Lever Products, Kolkata, India) and water. The experimental protocols were approved by the Institutional Ethical Committee of Panjab University, Chandigarh.

### Drugs

Chemicals employed in these studies were reagent grade. Carbon tetrachloride (E Merck, India) was administered subcutaneously in olive oil Hesperidin (Sigma chemical USA) was suspended in 0.5% sodium carboxy methyl cellulose (CMC) and administered orally.

### Experimental groups and protocol

Animals were divided into following groups, each containing 6–8 animals: *Control*: These animals received a vehicle for HDN (i.e. CMC) by oral route for eight days and on 8^th ^day, they were administered the subcutaneous injection of olive oil. *CCl_4 _group*: These animals received vehicle for 10 days and were challenged with CCl_4 _2 ml/kg/s.c. (40% v/v in olive oil) on 8^th ^day. In the preliminary studies done in our lab, we observed a very high mortality rate (50–60%) when CCl_4 _was administered interaperitoneally. Thus we adopted the subcutaneous route of CCl_4 _administration as reported in the literature [35]. With this route and dose of CCl_4_, the mortality rate reduced to about 20%(1-2/8 animal) HD*N group*: These rats received only HDN 200 mg/kg/p.o. daily for 10 days CCl_4_*+ HDN (100)*: Rats received HDN continuously for 8 days. On eight day just after HDN treatment they received CCl_4 _2 ml/kg/s.c in olive oil. HDN was further continued for 2 more days. CCl_4_*+ HDN (200)*: This group is similar to the above one except that the dose of HDN administered was 200 mg/kg/p.o. On the 10^th ^day, animals were sacrificed 2 hr, after the last dose of HDN and blood was collected, by carotid bleeding, in centrifuge tubes. Serum was separated and was used freshly for the assessment of renal and liver function tests. Both the kidneys and the liver were quickly harvested and immediately stored at -20°C till further biochemical estimations.

### Assessment of renal functions

Before sacrifice, rats were kept individually in metabolic cages for 24 h to collect urine for estimation of renal function. Serum samples were assayed for blood urea nitrogen (BUN), urea clearance, serum creatinine & creatinine clearance by using standard diagnostic kits (Span Diagnostics, Gujarat, India).

### Assessment of liver function

Serum alanine aminotransferase (ALT) and serum aspartate aminotransferase (AST) were estimated by International Federation of Clinical Chemistry [36] (ERBA test kits). Serum bilirubin was estimated by Diazo method [37] (ERBA test kits).

### Assessment of oxidative stress

#### Post mitochondrial supernatant preparation (PMS)

Kidneys and liver were, perfused with ice cold saline (0.9% sodium chloride) and homogenized in chilled potassium chloride (1.17%) using a homogenizer. The homogenates were centrifuged at 800 g for 5 minutes at 4°C to separate the nuclear debris. The supernatant so obtained was centrifuged at 10,500 g for 20 minutes at 4°C to get the post mitochondrial supernatant which was used to assay catalase and superoxide dismutase (SOD) activity.

#### Estimation of lipid peroxidation

The malondialdehyde (MDA) content, a measure of lipid peroxidation, was assayed in the form of thiobarbituric acid reacting substances (TBARS) by method of Okhawa et al. [38] Briefly, the reaction mixture consisted of 0.2 ml of 8.1% sodium lauryl sulphate, 1.5 ml of 20% acetic acid solution adjusted to pH 3.5 with sodium hydroxide and 1.5 ml of 0.8% aqueous solution of thiobarbituric acid was added to 0.2 ml of 10%(w/v) of PMS. The mixture was brought up to 4.0 ml with distilled water and heated at 95°C for 60 minutes. After cooling with tap water, 1.0 ml distilled water and 5.0 ml of the mixture of n-butanol & pyridine (15:1 v/v) was added and centrifuged. The organic layer was taken out and its absorbance was measured at 532 nm. TBARS were quantified using an extinction coefficient of 1.56 × 10^5 ^M^-1^/cm^-1 ^and expressed as nmol of TBARS per mg protein. Tissue protein was estimated using Biuret method of protein assay and the TBARS content expressed as nanomoles per milligram of protein.

#### Estimation of reduced glutathione

Reduced glutathione (GSH) in the kidneys and liver was assayed by the method of Jollow et al [39]. Briefly, 1.0 ml of PMS (10%) was precipitated with 1.0 ml of sulphosalicylic acid (4%). The samples were kept at 4°C for at least 1 hour and then subjected to centrifugation at 1200 g for 15 minutes at 4°C. The assay mixture contained 0.1 ml filtered aliquot and 2.7 ml phosphate buffer (0.1 M, pH 7.4) in a total volume of 3.0 ml. The yellow colour developed was read immediately at 412 nm on a spectrophotometer.

#### Estimation of SOD

SOD activity was assayed by the method of Kono et al.[40] The assay system consisted of EDTA 0.1 mM, sodium carbonate 50 mM and 96 mM of nitro blue tetrazolium (NBT). In the cuvette, 2 ml of above mixture, 0.05 ml hydroxylamine and 0.05 ml of PMS were taken and the auto-oxidation of hydroxylamine was observed by measuring the absorbance at 560 nm.

#### Estimation of catalase

Catalase activity was assayed by the method of Claiborne et al [41]. Briefly, the assay mixture consisted of 1.95 ml phosphate buffer (0.05 M, pH 7.0), 1.0 ml hydrogen peroxide (0.019 M) and 0.05 ml PMS (10%) in a final volume of 3.0 ml. Changes in absorbance were recorded at 240 nm. Catalase activity was calculated in terms of k minutes^-1^.

### Statistical analysis

Results were expressed as mean ± SEM. The intergroup variation was measured by one way analysis of variance (ANOVA) followed by Fischer's LSD test. Statistical significance was considered at p < 0.05. The statistical analysis was done using the Jandel Sigma Stat Statistical Software version 2.0.

## Authors' contributions

Naveen Tirkey, Sangeeta Pilkhwal and Anurag did all the biochemical estimations in kidney and liver. Kanwaljit Chopra did the data interpretation after statistical analysis and contributed in manuscript preparation.

## References

[B1] Abraham P, Wilfred G, Cathrine (1999). Oxidative damage to the lipids and proteins pf the lungs, testis and kidney of rats during carbon tetrachloride intoxication. Clin Chim Acta.

[B2] Szymonik-Lesiuk S, Czechowska G, Stryjecka-Zimmer M, Slomka M, Madro A, Celinski K, Wielosz M (2003). Catalase, superoxide dismutase, and glutathione peroxidase activities in various rat tissues after carbon tetrachloride intoxication.. J Hepatobiliary Pancreat Surg.

[B3] Guven A, Guven A, Gulmez M (2003). The effect of kefir on the activities of GSH-Px, GST, CAT, GSH and LPO levels in carbon tetrachloride-induced mice tissue. J Vet Med B Infect Dis Vet Public Health.

[B4] Ahmad FF, Cowan DL, Sun AY (1987). Detection of free radical formation in various tissues after acute carbon tetrachloride administration in gerbil. Life Sci.

[B5] Ohta Y, Nishida K, Sasaki E, Kongo M, Ishiguro I (1997). Attenuation of disrupted hepatic active oxygen metabolism with the recovery of acute liver injury in rats intoxicated with carbon tetrachloride. Res Commun Mol Pathol Pharmacol.

[B6] Ozturk F, Ucar M, Ozturk IC, Vardi N, Batcioglu K (2003). Carbon tetrachloride-induced nephrotoxicity and protective effect of betaine in Sprague-Dawley rats. Urology.

[B7] Perez AJ, Courel M, Sobrado J, Gonzalez L (1987). Acute renal failure after topical application of carbon tetrachloride. Lancet.

[B8] Churchill DN, Finn A, Gault M (1983). Association between hydrocharbon exposure and glomerulonephritis.An araisal of the evidence. Nephron.

[B9] Ruprah H, Mant TGK, Flanagan RJ (1985). Acute carbon tetrachloride poisoning in19 pateints: implications for diagnosis and treatment. Lancet.

[B10] Yoshikawa T, Furukawa Y, Murakami M, Takemura S, Kondo M (1982). Effect of viatmin E on D-Galactosamine-induced or carbon tetrachloride-induced hepatotoxicity. Digestion.

[B11] Ko KM, Ip SP, Poon MK, Wu SS, Che CT, Ng KH, Kong YC (1995). Effect of a lignan-enriched fructus schisandrae extract on hepatic glutathione status in rats: protection against carbon tetrachloride toxicity. Planta Med.

[B12] Garg A, Garg S, Zaneveld LJ, Singla AK (2001). Chemistry and pharmacology of the Citrus bioflavonoid hesperidin. Phytother Res.

[B13] Jovanovic SV, Steenken S, Tosic M, Marjanovic B, Simic MG (1994). Flavonoids as anti-oxidants. J Am Chem Soc.

[B14] Fraga CG, Martino VS, Ferraro GE, Coussio JD, Boveris A (1987). Flavonoids as antioxidants evaluated by in vitro and in situ liver chemiluminescence. Biochem Pharmacol.

[B15] Miller NJ, Rice-Evans CA (1997). The relative contribution of ascorbic acid and phenolic antioxidants to the total antioxidant activity of orange and apple fruit juices and balckcurrant drink. Food Chem.

[B16] Suarez, Herrera MD, Marhuenda E (1998). In vitro scavenger and antioxidant properites of hesperidin and neohesperidin dihydrochalcone. Phytomedicine.

[B17] Recknagel RO (1983). Carbon tetrachloride hepatotoxicity: Status quo and future prospects. TIPS.

[B18] Recknagel RO, Ghoshal AK (1966). Lipoperoxidation as a vector in carbon tetrachloride hepatotoxicity. Lab Invest.

[B19] Durk H, Frank H (1984). Carbon tetrachloride metabolism in vivo and exhalation of volatile alkanes: dependence upon oxygen parital pressure.. Toxicology.

[B20] Mansuy D, Fontecane M, Chottard J (1980). A heme model study of carbon tetrachloride metabolism: mechanism of phosgene and carbon dioxide formation. Biochem Biophys Res Commun.

[B21] Pohl L, Schulick R, George J (1984). Reductive oxygenation mechanism of metabolism of carbon tetrachlorideto phosgene by cytochrome P450. Mol Pharmacol.

[B22] Pohl L, Schulick R, Highet R, George J (1983). Identification of dichloromethyl carbene as a metabolite of carbon tetrachloride. Biochem Biophys Res Commun.

[B23] Tom WM, Fong D, Woo D, Prasongwatana V, Boyde TR (1984). Microsomal lipid peroxidation and oxidative metabolism in rat liver. Chem Biol Interact.

[B24] Slater TF (1984). Free-radical mechanisms in tissue injury. Biochem J.

[B25] Recknagel RO, Glende EA, Lowery K, Plaa GL and Hewitt W (1982). Lipid peroxidation: Biochemisry, measurement and significance in liver cell injury. Toxicology of the Liver.

[B26] ElSisi AE, Earnest DL, Sipes IG (1993). Vitamin A potentiation of carbon tetrachloride hepatotoxicity: role of liver macrophages and active oxygen species. Toxicol Appl Pharmacol.

[B27] Hsiao G, Shen MY, Lin KH, Lan MH, Wu LY, Chou DS, Lin CH, Su CH, Sheu JR (2003). Antioxidative and hepatoprotective effects of Antrodia camphorata extract. J Agric Food Chem.

[B28] Jeong TC, Kim HJ, Park J, Ha CS, Park JD, Kim S, Roh JK (1996). Protective effects of red ginseng saponins against carbon tetrachloride-induced hepatotoxicity in sprague dawley rats. Planta Med.

[B29] Zimmerman SW, Norback DH, Powers K (1983). Carbon tetrachloride nephrotoxicity in rats with reduced renal mass. Arch Pathol Lab Med.

[B30] Ogawa M, Mori T, Mori Y, Ueda S, Azemoto R, Makino Y, Wakashin Y, Ohto M, Wakashin M, Yoshida H (1992). Study on chronic renal injuries induced by carbon tetrachloride: selective inhibition of the nephrotoxicity by irradiation. Nephron.

[B31] Fadhel ZA, Amran S (2002). Effects of black tea extract on carbon tetrachloride-induced lipid peroxidation in liver, kidneys, and testes of rats. Phytother Res.

[B32] Patil S, Kanase A, Varute AT (1989). Effect of hepatoprotective ayurvedic drugs on lipases following CCl4  induced hepatic injury in rats. Indian J Exp Biol.

[B33] Miyake Y, Yamamoto K, Tsujihara N, Osawa T (1998). Protective effects of lemon flavonoids on oxidative stress in diabetic rats. Lipids.

[B34] Jean T, Bodinier MC (1994). Mediators involved in inflammation: effects of Daflon 500 mg on their release. Angiology.

[B35] Mandal AK, Sinha J, Mandal S, Mukhopadhyay S, Das N (2002). Targeting of liposomal flavonoid to liver in combating hepatocellular oxidative damage. Drug Deliv.

[B36] (1980). IFCC methods for the measurement of catalytic concentrations of enzymes. part 3, IFCC. Method for alanine aminotransferase (l-alanine 2 -oxoglutarate aminotransferase, ec 2.6.1.2).. Clin Chim Acta.

[B37] Pearlman FC, Lee RT (1974). Detection and measurement of total bilirubin in serum, with use of surfactants as solubilizing agents. Clin Chem.

[B38] Ohkawa H, Ohishi N, Yagi K (1979). Assay for lipid peroxides in animal tissues by thiobarbituric acid reaction. Anal Biocem.

[B39] Jollow D, Mitchell L, Zampaglione N, Gillete J (1974). Bromobenze induced liver necrosis: protective role of glutathione and evidence for 3,4-bromobenzenoxide as the hepatotoxic intermediate. Pharmacol.

[B40] Kono Y (1978). Generation of superoxide radical during autoxidation of hydroxylamine and an assay for superoxide dismutase. Arch Biochem Biophys.

[B41] Claiborne A, Boca Raton FL (1985). Handbook of Methods for Oxygen Radical Reaserch.

